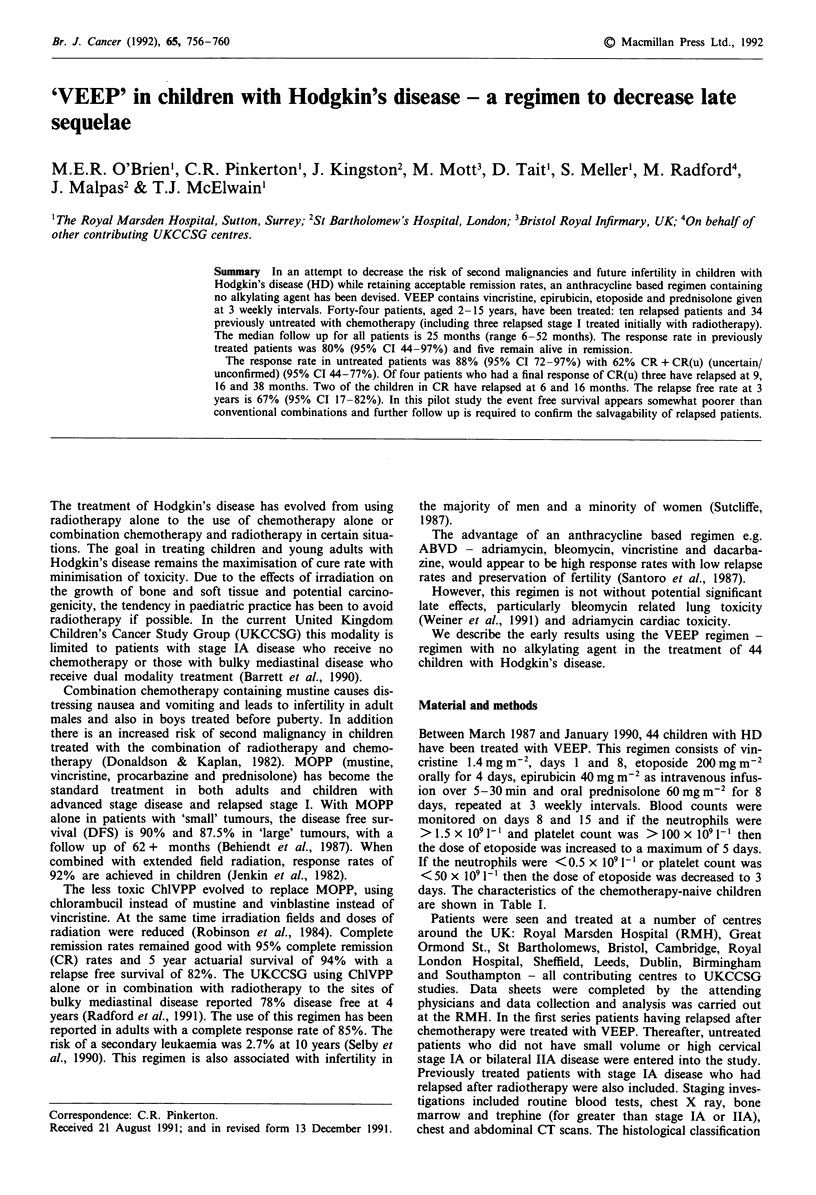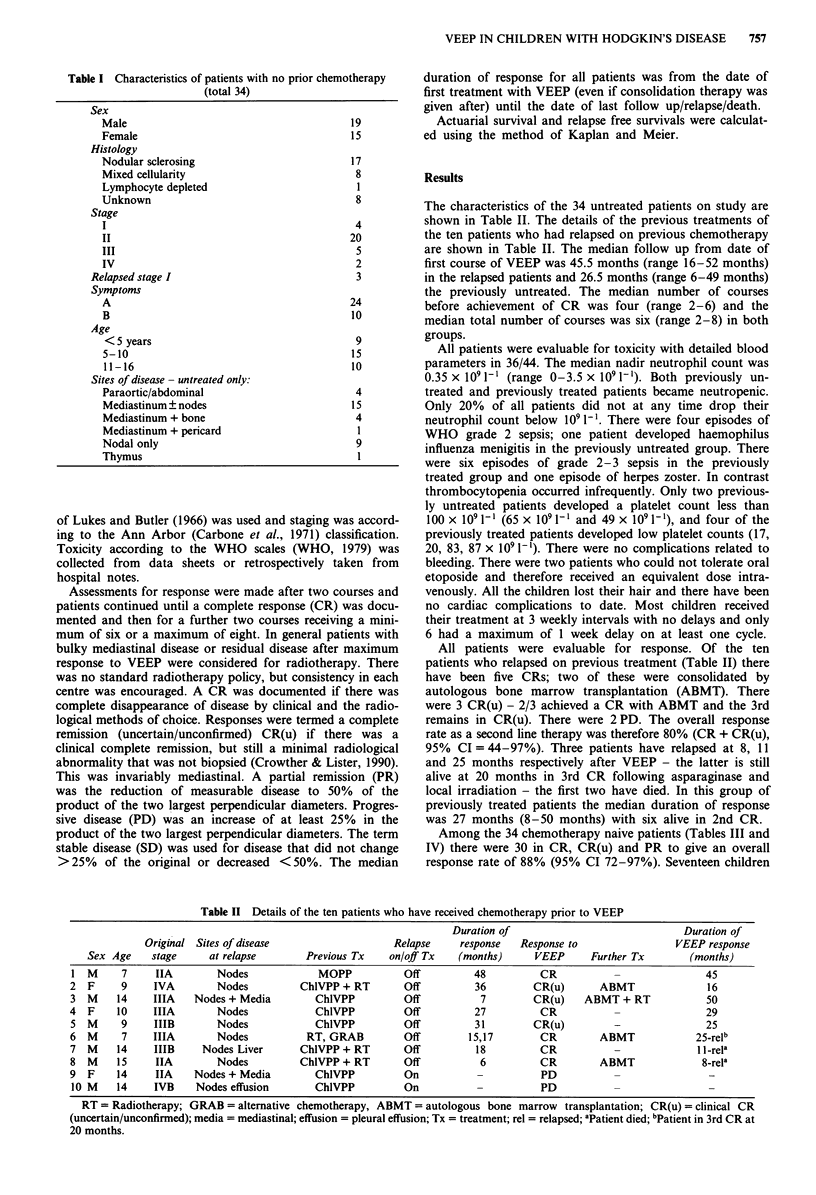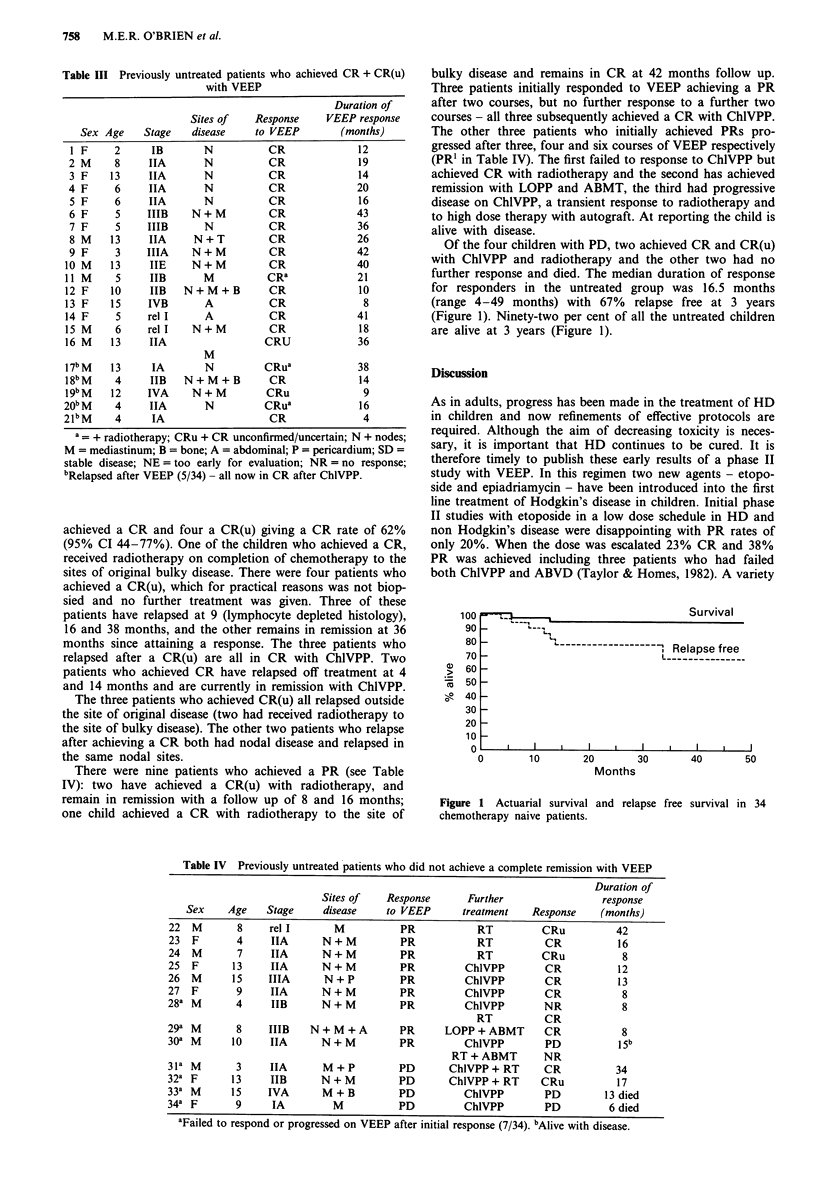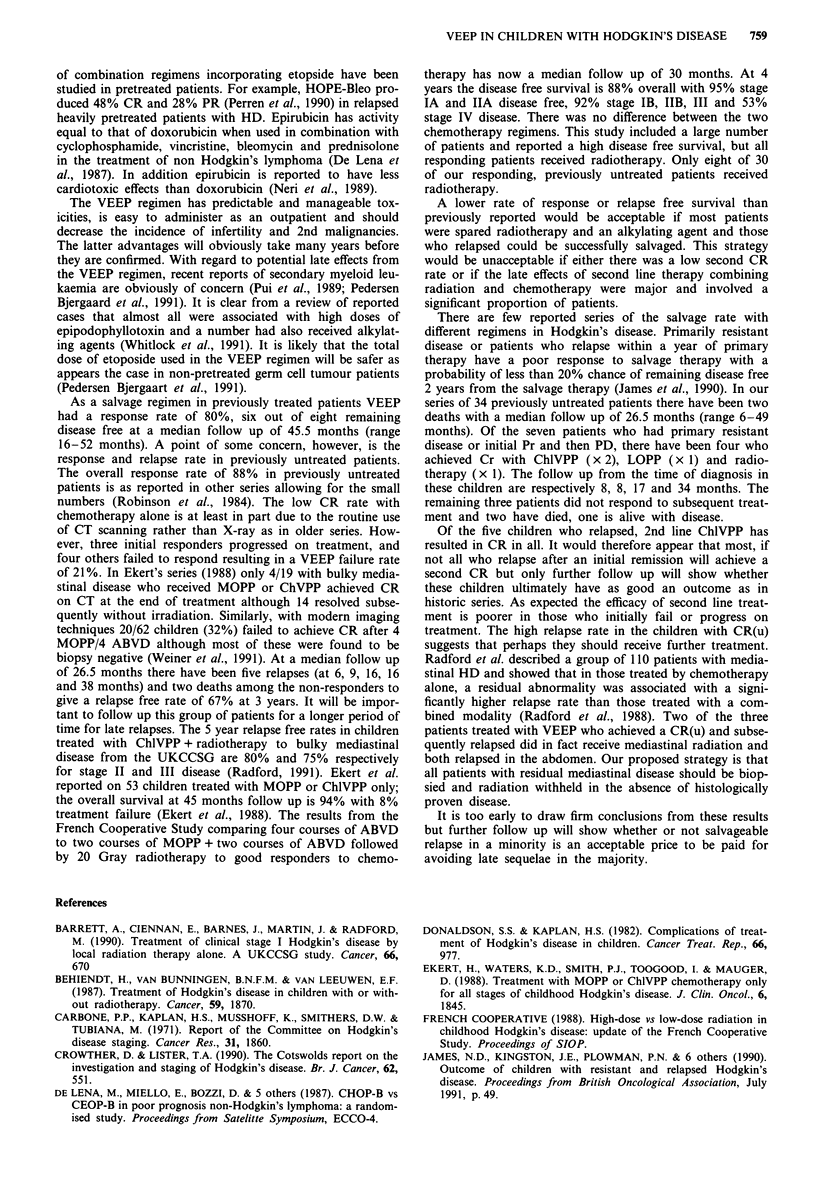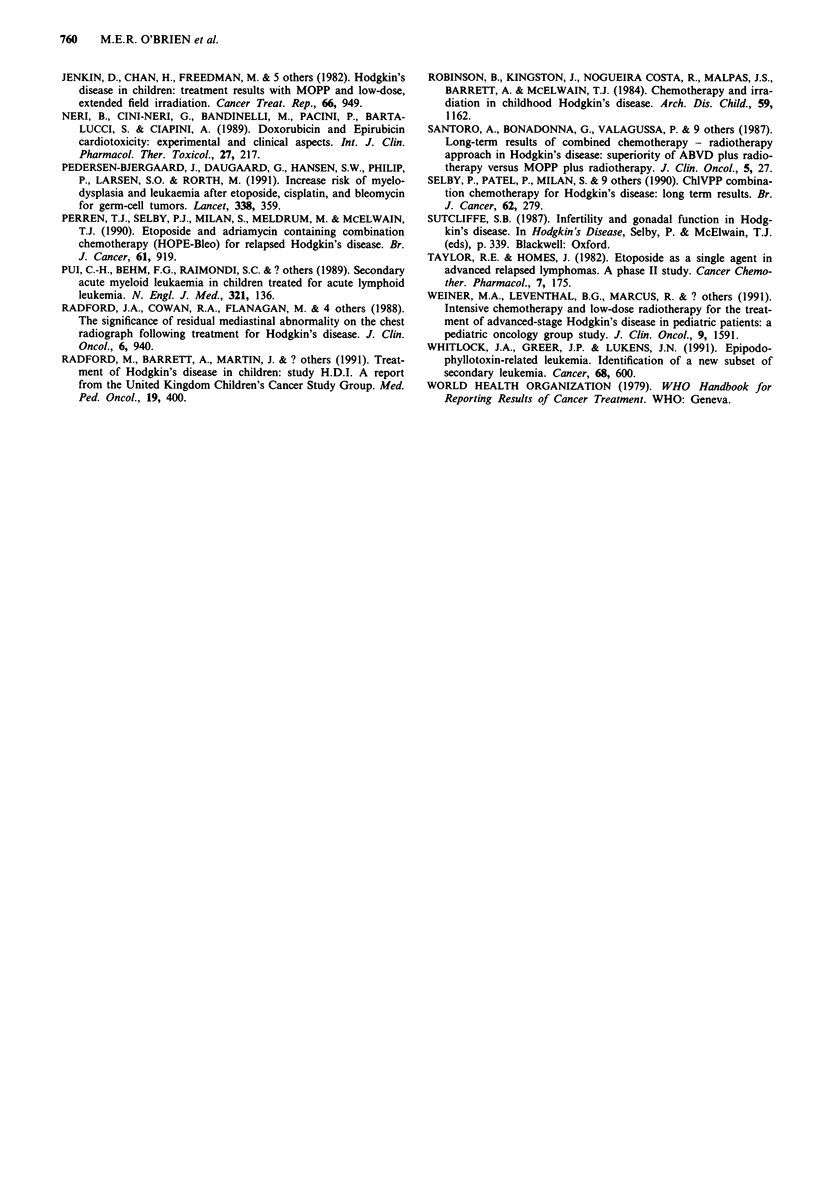# 'VEEP' in children with Hodgkin's disease--a regimen to decrease late sequelae.

**DOI:** 10.1038/bjc.1992.159

**Published:** 1992-05

**Authors:** M. E. O'Brien, C. R. Pinkerton, J. Kingston, M. Mott, D. Tait, S. Meller, M. Radford, J. Malpas, T. J. McElwain

**Affiliations:** Royal Marsden Hospital, Sutton, Surrey, UK.

## Abstract

In an attempt to decrease the risk of second malignancies and future infertility in children with Hodgkin's disease (HD) while retaining acceptable remission rates, an anthracycline based regimen containing no alkylating agent has been devised. VEEP contains vincristine, epirubicin, etoposide and prednisolone given at 3 weekly intervals. Forty-four patients, aged 2-15 years, have been treated: ten relapsed patients and 34 previously untreated with chemotherapy (including three relapsed stage I treated initially with radiotherapy). The median follow up for all patients is 25 months (range 6-52 months). The response rate in previously treated patients was 80% (95% CI 44-97%) and five remain alive in remission. The response rate in untreated patients was 88% (95% CI 72-97%) with 62% CR + CR(u) (uncertain/unconfirmed) (95% CI 44-77%). Of four patients who had a final response of CR(u) three have relapsed at 9, 16 and 38 months. Two of the children in CR have relapsed at 6 and 16 months. The relapse free rate at 3 years is 67% (95% CI 17-82%). In this pilot study the event free survival appears somewhat poorer than conventional combinations and further follow up is required to confirm the salvagability of relapsed patients.


Br. J. Cancer (1992), 65, 756-760 © Macmillan Press Ltd., 1992

### 'VEEP' in children with Hodgkin's disease – a regimen to decrease late sequelae

M.E.R. O'Brien[1], C.R. Pinkerton[1], J. Kingston[2], M. Mott[3], D. Tait[1], S. Meller[1], M. Radford[4], J. Malpas[2] & T.J. McElwain[1]

[1]*The Royal Marsden Hospital, Sutton, Surrey;* [2]*St Bartholomew's Hospital, London;* [3]*Bristol Royal Infirmary, UK;* [4]*On behalf of other contributing UKCCSG centres.*

**Summary** In an attempt to decrease the risk of second malignancies and future infertility in children with Hodgkin's disease (HD) while retaining acceptable remission rates, an anthracycline based regimen containing no alkylating agent has been devised. VEEP contains vincristine, epirubicin, etoposide and prednisolone given at 3 weekly intervals. Forty-four patients, aged 2–15 years, have been treated: ten relapsed patients and 34 previously untreated with chemotherapy (including three relapsed stage I treated initially with radiotherapy). The median follow up for all patients is 25 months (range 6–52 months). The response rate in previously treated patients was 80% (95% CI 44–97%) and five remain alive in remission.

The response rate in untreated patients was 88% (95% CI 72–97%) with 62% CR + CR(u) (uncertain/unconfirmed) (95% CI 44–77%). Of four patients who had a final response of CR(u) three have relapsed at 9, 16 and 38 months. Two of the children in CR have relapsed at 6 and 16 months. The relapse free rate at 3 years is 67% (95% CI 17–82%). In this pilot study the event free survival appears somewhat poorer than conventional combinations and further follow up is required to confirm the salvagability of relapsed patients.

The treatment of Hodgkin's disease has evolved from using radiotherapy alone to the use of chemotherapy alone or combination chemotherapy and radiotherapy in certain situations. The goal in treating children and young adults with Hodgkin's disease remains the maximisation of cure rate with minimisation of toxicity. Due to the effects of irradiation on the growth of bone and soft tissue and potential carcinogenicity, the tendency in paediatric practice has been to avoid radiotherapy if possible. In the current United Kingdom Children's Cancer Study Group (UKCCSG) this modality is limited to patients with stage IA disease who receive no chemotherapy or those with bulky mediastinal disease who receive dual modality treatment (Barrett *et al.*, 1990).

Combination chemotherapy containing mustine causes distressing nausea and vomiting and leads to infertility in adult males and also in boys treated before puberty. In addition there is an increased risk of second malignancy in children treated with the combination of radiotherapy and chemotherapy (Donaldson & Kaplan, 1982). MOPP (mustine, vincristine, procarbazine and prednisolone) has become the standard treatment in both adults and children with advanced stage disease and relapsed stage I. With MOPP alone in patients with 'small' tumours, the disease free survival (DFS) is 90% and 87.5% in 'large' tumours, with a follow up of 62+ months (Behiendt *et al.*, 1987). When combined with extended field radiation, response rates of 92% are achieved in children (Jenkin *et al.*, 1982).

The less toxic ChlVPP evolved to replace MOPP, using chlorambucil instead of mustine and vinblastine instead of vincristine. At the same time irradiation fields and doses of radiation were reduced (Robinson *et al.*, 1984). Complete remission rates remained good with 95% complete remission (CR) rates and 5 year actuarial survival of 94% with a relapse free survival of 82%. The UKCCSG using ChlVPP alone or in combination with radiotherapy to the sites of bulky mediastinal disease reported 78% disease free at 4 years (Radford *et al.*, 1991). The use of this regimen has been reported in adults with a complete response rate of 85%. The risk of a secondary leukaemia was 2.7% at 10 years (Selby *et al.*, 1990). This regimen is also associated with infertility in the majority of men and a minority of women (Sutcliffe, 1987).

The advantage of an anthracycline based regimen e.g. ABVD – adriamycin, bleomycin, vincristine and dacarbazine, would appear to be high response rates with low relapse rates and preservation of fertility (Santoro *et al.*, 1987).

However, this regimen is not without potential significant late effects, particularly bleomycin related lung toxicity (Weiner *et al.*, 1991) and adriamycin cardiac toxicity.

We describe the early results using the VEEP regimen – regimen with no alkylating agent in the treatment of 44 children with Hodgkin's disease.

### Material and methods

Between March 1987 and January 1990, 44 children with HD have been treated with VEEP. This regimen consists of vincristine 1.4 mg $m^{-2}$, days 1 and 8, etoposide 200 mg $m^{-2}$ orally for 4 days, epirubicin 40 mg $m^{-2}$ as intravenous infusion over 5–30 min and oral prednisolone 60 mg $m^{-2}$ for 8 days, repeated at 3 weekly intervals. Blood counts were monitored on days 8 and 15 and if the neutrophils were $> 1.5 \times 10^9 l^{-1}$ and platelet count was $> 100 \times 10^9 l^{-1}$ then the dose of etoposide was increased to a maximum of 5 days. If the neutrophils were $< 0.5 \times 10^9 l^{-1}$ or platelet count was $< 50 \times 10^9 l^{-1}$ then the dose of etoposide was decreased to 3 days. The characteristics of the chemotherapy-naive children are shown in Table I.

Patients were seen and treated at a number of centres around the UK: Royal Marsden Hospital (RMH), Great Ormond St., St Bartholomews, Bristol, Cambridge, Royal London Hospital, Sheffield, Leeds, Dublin, Birmingham and Southampton – all contributing centres to UKCCSG studies. Data sheets were completed by the attending physicians and data collection and analysis was carried out at the RMH. In the first series patients having relapsed after chemotherapy were treated with VEEP. Thereafter, untreated patients who did not have small volume or high cervical stage IA or bilateral IIA disease were entered into the study. Previously treated patients with stage IA disease who had relapsed after radiotherapy were also included. Staging investigations included routine blood tests, chest X ray, bone marrow and trephine (for greater than stage IA or IIA), chest and abdominal CT scans. The histological classification

Correspondence: C.R. Pinkerton.
Received 21 August 1991; and in revised form 13 December 1991.

**Table I** Characteristics of patients with no prior chemotherapy (total 34)

| | |
|---|---|
| *Sex* | |
| Male | 19 |
| Female | 15 |
| *Histology* | |
| Nodular sclerosing | 17 |
| Mixed cellularity | 8 |
| Lymphocyte depleted | 1 |
| Unknown | 8 |
| *Stage* | |
| I | 4 |
| II | 20 |
| III | 5 |
| IV | 2 |
| *Relapsed stage I* | 3 |
| *Symptoms* | |
| A | 24 |
| B | 10 |
| *Age* | |
| <5 years | 9 |
| 5–10 | 15 |
| 11–16 | 10 |
| *Sites of disease – untreated only:* | |
| Paraortic/abdominal | 4 |
| Mediastinum ± nodes | 15 |
| Mediastinum + bone | 4 |
| Mediastinum + pericard | 1 |
| Nodal only | 9 |
| Thymus | 1 |

of Lukes and Butler (1966) was used and staging was according to the Ann Arbor (Carbone *et al.*, 1971) classification. Toxicity according to the WHO scales (WHO, 1979) was collected from data sheets or retrospectively taken from hospital notes.

Assessments for response were made after two courses and patients continued until a complete response (CR) was documented and then for a further two courses receiving a minimum of six or a maximum of eight. In general patients with bulky mediastinal disease or residual disease after maximum response to VEEP were considered for radiotherapy. There was no standard radiotherapy policy, but consistency in each centre was encouraged. A CR was documented if there was complete disappearance of disease by clinical and the radiological methods of choice. Responses were termed a complete remission (uncertain/unconfirmed) CR(u) if there was a clinical complete remission, but still a minimal radiological abnormality that was not biopsied (Crowther & Lister, 1990). This was invariably mediastinal. A partial remission (PR) was the reduction of measurable disease to 50% of the product of the two largest perpendicular diameters. Progressive disease (PD) was an increase of at least 25% in the product of the two largest perpendicular diameters. The term stable disease (SD) was used for disease that did not change >25% of the original or decreased <50%. The median duration of response for all patients was from the date of first treatment with VEEP (even if consolidation therapy was given after) until the date of last follow up/relapse/death.

Actuarial survival and relapse free survivals were calculated using the method of Kaplan and Meier.

### Results

The characteristics of the 34 untreated patients on study are shown in Table II. The details of the previous treatments of the ten patients who had relapsed on previous chemotherapy are shown in Table II. The median follow up from date of first course of VEEP was 45.5 months (range 16–52 months) in the relapsed patients and 26.5 months (range 6–49 months) the previously untreated. The median number of courses before achievement of CR was four (range 2–6) and the median total number of courses was six (range 2–8) in both groups.

All patients were evaluable for toxicity with detailed blood parameters in 36/44. The median nadir neutrophil count was $0.35 \times 10^9 l^{-1}$ (range $0-3.5 \times 10^9 l^{-1}$). Both previously untreated and previously treated patients became neutropenic. Only 20% of all patients did not at any time drop their neutrophil count below $10^9 l^{-1}$. There were four episodes of WHO grade 2 sepsis; one patient developed haemophilus influenza menigitis in the previously untreated group. There were six episodes of grade 2–3 sepsis in the previously treated group and one episode of herpes zoster. In contrast thrombocytopenia occurred infrequently. Only two previously untreated patients developed a platelet count less than $100 \times 10^9 l^{-1}$ ($65 \times 10^9 l^{-1}$ and $49 \times 10^9 l^{-1}$), and four of the previously treated patients developed low platelet counts (17, 20, 83, $87 \times 10^9 l^{-1}$). There were no complications related to bleeding. There were two patients who could not tolerate oral etoposide and therefore received an equivalent dose intravenously. All the children lost their hair and there have been no cardiac complications to date. Most children received their treatment at 3 weekly intervals with no delays and only 6 had a maximum of 1 week delay on at least one cycle.

All patients were evaluable for response. Of the ten patients who relapsed on previous treatment (Table II) there have been five CRs; two of these were consolidated by autologous bone marrow transplantation (ABMT). There were 3 CR(u) – 2/3 achieved a CR with ABMT and the 3rd remains in CR(u). There were 2 PD. The overall response rate as a second line therapy was therefore 80% (CR + CR(u), 95% CI = 44–97%). Three patients have relapsed at 8, 11 and 25 months respectively after VEEP – the latter is still alive at 20 months in 3rd CR following asparaginase and local irradiation – the first two have died. In this group of previously treated patients the median duration of response was 27 months (8–50 months) with six alive in 2nd CR.

Among the 34 chemotherapy naive patients (Tables III and IV) there were 30 in CR, CR(u) and PR to give an overall response rate of 88% (95% CI 72–97%). Seventeen children

**Table II** Details of the ten patients who have received chemotherapy prior to VEEP

| | Sex | Age | *Original stage* | *Sites of disease at relapse* | *Previous Tx* | *Relapse on/off Tx* | *Duration of response (months)* | *Response to VEEP* | *Further Tx* | *Duration of VEEP response (months)* |
|---|---|---|---|---|---|---|---|---|---|---|
| 1 | M | 7 | IIA | Nodes | MOPP | Off | 48 | CR | – | 45 |
| 2 | F | 9 | IVA | Nodes | ChlVPP + RT | Off | 36 | CR(u) | ABMT | 16 |
| 3 | M | 14 | IIIA | Nodes + Media | ChlVPP | Off | 7 | CR(u) | ABMT + RT | 50 |
| 4 | F | 10 | IIIA | Nodes | ChlVPP | Off | 27 | CR | – | 29 |
| 5 | M | 9 | IIIB | Nodes | ChlVPP | Off | 31 | CR(u) | – | 25 |
| 6 | M | 7 | IIIA | Nodes | RT, GRAB | Off | 15,17 | CR | ABMT | 25-rel[b] |
| 7 | M | 14 | IIIB | Nodes Liver | ChlVPP + RT | Off | 18 | CR | – | 11-rel[a] |
| 8 | M | 15 | IIA | Nodes | ChlVPP + RT | Off | 6 | CR | ABMT | 8-rel[a] |
| 9 | F | 14 | IIA | Nodes + Media | ChlVPP | On | – | PD | – | – |
| 10 | M | 14 | IVB | Nodes effusion | ChlVPP | On | – | PD | – | – |

RT = Radiotherapy; GRAB = alternative chemotherapy, ABMT = autologous bone marrow transplantation; CR(u) = clinical CR (uncertain/unconfirmed); media = mediastinal; effusion = pleural effusion; Tx = treatment; rel = relapsed; [a]Patient died; [b]Patient in 3rd CR at 20 months.

**Table III** Previously untreated patients who achieved CR + CR(u) with VEEP

| | Sex | Age | Stage | Sites of disease | Response to VEEP | Duration of VEEP response (months) |
|---|---|---|---|---|---|---|
| 1 | F | 2 | IB | N | CR | 12 |
| 2 | M | 8 | IIA | N | CR | 19 |
| 3 | F | 13 | IIA | N | CR | 14 |
| 4 | F | 6 | IIA | N | CR | 20 |
| 5 | F | 6 | IIA | N | CR | 16 |
| 6 | F | 5 | IIIB | N + M | CR | 43 |
| 7 | F | 5 | IIIB | N | CR | 36 |
| 8 | M | 13 | IIA | N + T | CR | 26 |
| 9 | F | 3 | IIIA | N + M | CR | 42 |
| 10 | M | 13 | IIE | N + M | CR | 40 |
| 11 | M | 5 | IIB | M | CR[a] | 21 |
| 12 | F | 10 | IIB | N + M + B | CR | 10 |
| 13 | F | 15 | IVB | A | CR | 8 |
| 14 | F | 5 | rel I | A | CR | 41 |
| 15 | M | 6 | rel I | N + M | CR | 18 |
| 16 | M | 13 | IIA | | CRU | 36 |
| | | | | M | | |
| 17[b] | M | 13 | IA | N | CRu[a] | 38 |
| 18[b] | M | 4 | IIB | N + M + B | CR | 14 |
| 19[b] | M | 12 | IVA | N + M | CRu | 9 |
| 20[b] | M | 4 | IIA | N | CRu[a] | 16 |
| 21[b] | M | 4 | IA | | CR | 4 |

[a] = + radiotherapy; CRu + CR unconfirmed/uncertain; N + nodes; M = mediastinum; B = bone; A = abdominal; P = pericardium; SD = stable disease; NE = too early for evaluation; NR = no response; [b]Relapsed after VEEP (5/34) – all now in CR after ChlVPP.

achieved a CR and four a CR(u) giving a CR rate of 62% (95% CI 44–77%). One of the children who achieved a CR, received radiotherapy on completion of chemotherapy to the sites of original bulky disease. There were four patients who achieved a CR(u), which for practical reasons was not biopsied and no further treatment was given. Three of these patients have relapsed at 9 (lymphocyte depleted histology), 16 and 38 months, and the other remains in remission at 36 months since attaining a response. The three patients who relapsed after a CR(u) are all in CR with ChlVPP. Two patients who achieved CR have relapsed off treatment at 4 and 14 months and are currently in remission with ChlVPP.

The three patients who achieved CR(u) all relapsed outside the site of original disease (two had received radiotherapy to the site of bulky disease). The other two patients who relapse after achieving a CR both had nodal disease and relapsed in the same nodal sites.

There were nine patients who achieved a PR (see Table IV): two have achieved a CR(u) with radiotherapy, and remain in remission with a follow up of 8 and 16 months; one child achieved a CR with radiotherapy to the site of bulky disease and remains in CR at 42 months follow up. Three patients initially responded to VEEP achieving a PR after two courses, but no further response to a further two courses – all three subsequently achieved a CR with ChlVPP. The other three patients who initially achieved PRs progressed after three, four and six courses of VEEP respectively (PR[1] in Table IV). The first failed to response to ChlVPP but achieved CR with radiotherapy and the second has achieved remission with LOPP and ABMT, the third had progressive disease on ChlVPP, a transient response to radiotherapy and to high dose therapy with autograft. At reporting the child is alive with disease.

Of the four children with PD, two achieved CR and CR(u) with ChlVPP and radiotherapy and the other two had no further response and died. The median duration of response for responders in the untreated group was 16.5 months (range 4–49 months) with 67% relapse free at 3 years (Figure 1). Ninety-two per cent of all the untreated children are alive at 3 years (Figure 1).

### Discussion

As in adults, progress has been made in the treatment of HD in children and now refinements of effective protocols are required. Although the aim of decreasing toxicity is necessary, it is important that HD continues to be cured. It is therefore timely to publish these early results of a phase II study with VEEP. In this regimen two new agents – etoposide and epiadriamycin – have been introduced into the first line treatment of Hodgkin's disease in children. Initial phase II studies with etoposide in a low dose schedule in HD and non Hodgkin's disease were disappointing with PR rates of only 20%. When the dose was escalated 23% CR and 38% PR was achieved including three patients who had failed both ChlVPP and ABVD (Taylor & Homes, 1982). A variety

**Figure 1** Actuarial survival and relapse free survival in 34 chemotherapy naive patients.

**Table IV** Previously untreated patients who did not achieve a complete remission with VEEP

| | Sex | Age | Stage | Sites of disease | Response to VEEP | Further treatment | Response | Duration of response (months) |
|---|---|---|---|---|---|---|---|---|
| 22 | M | 8 | rel I | M | PR | RT | CRu | 42 |
| 23 | F | 4 | IIA | N + M | PR | RT | CR | 16 |
| 24 | M | 7 | IIA | N + M | PR | RT | CRu | 8 |
| 25 | F | 13 | IIA | N + M | PR | ChlVPP | CR | 12 |
| 26 | M | 15 | IIIA | N + P | PR | ChlVPP | CR | 13 |
| 27 | F | 9 | IIA | N + M | PR | ChlVPP | CR | 8 |
| 28[a] | M | 4 | IIB | N + M | PR | ChlVPP | NR | 8 |
| | | | | | | RT | CR | |
| 29[a] | M | 8 | IIIB | N + M + A | PR | LOPP + ABMT | CR | 8 |
| 30[a] | M | 10 | IIA | N + M | PR | ChlVPP | PD | 15[b] |
| | | | | | | RT + ABMT | NR | |
| 31[a] | M | 3 | IIA | M + P | PD | ChlVPP + RT | CR | 34 |
| 32[a] | F | 13 | IIB | N + M | PD | ChlVPP + RT | CRu | 17 |
| 33[a] | M | 15 | IVA | M + B | PD | ChlVPP | PD | 13 died |
| 34[a] | F | 9 | IA | M | PD | ChlVPP | PD | 6 died |

[a]Failed to respond or progressed on VEEP after initial response (7/34). [b]Alive with disease.

of combination regimens incorporating etopside have been studied in pretreated patients. For example, HOPE-Bleo produced 48% CR and 28% PR (Perren *et al.*, 1990) in relapsed heavily pretreated patients with HD. Epirubicin has activity equal to that of doxorubicin when used in combination with cyclophosphamide, vincristine, bleomycin and prednisolone in the treatment of non Hodgkin's lymphoma (De Lena *et al.*, 1987). In addition epirubicin is reported to have less cardiotoxic effects than doxorubicin (Neri *et al.*, 1989).

The VEEP regimen has predictable and manageable toxicities, is easy to administer as an outpatient and should decrease the incidence of infertility and 2nd malignancies. The latter advantages will obviously take many years before they are confirmed. With regard to potential late effects from the VEEP regimen, recent reports of secondary myeloid leukaemia are obviously of concern (Pui *et al.*, 1989; Pedersen Bjergaard *et al.*, 1991). It is clear from a review of reported cases that almost all were associated with high doses of epipodophyllotoxin and a number had also received alkylating agents (Whitlock *et al.*, 1991). It is likely that the total dose of etoposide used in the VEEP regimen will be safer as appears the case in non-pretreated germ cell tumour patients (Pedersen Bjergaart *et al.*, 1991).

As a salvage regimen in previously treated patients VEEP had a response rate of 80%, six out of eight remaining disease free at a median follow up of 45.5 months (range 16–52 months). A point of some concern, however, is the response and relapse rate in previously untreated patients. The overall response rate of 88% in previously untreated patients is as reported in other series allowing for the small numbers (Robinson *et al.*, 1984). The low CR rate with chemotherapy alone is at least in part due to the routine use of CT scanning rather than X-ray as in older series. However, three initial responders progressed on treatment, and four others failed to respond resulting in a VEEP failure rate of 21%. In Ekert's series (1988) only 4/19 with bulky mediastinal disease who received MOPP or ChVPP achieved CR on CT at the end of treatment although 14 resolved subsequently without irradiation. Similarly, with modern imaging techniques 20/62 children (32%) failed to achieve CR after 4 MOPP/4 ABVD although most of these were found to be biopsy negative (Weiner *et al.*, 1991). At a median follow up of 26.5 months there have been five relapses (at 6, 9, 16, 16 and 38 months) and two deaths among the non-responders to give a relapse free rate of 67% at 3 years. It will be important to follow up this group of patients for a longer period of time for late relapses. The 5 year relapse free rates in children treated with ChlVPP + radiotherapy to bulky mediastinal disease from the UKCCSG are 80% and 75% respectively for stage II and III disease (Radford, 1991). Ekert *et al.* reported on 53 children treated with MOPP or ChlVPP only; the overall survival at 45 months follow up is 94% with 8% treatment failure (Ekert *et al.*, 1988). The results from the French Cooperative Study comparing four courses of ABVD to two courses of MOPP + two courses of ABVD followed by 20 Gray radiotherapy to good responders to chemotherapy has now a median follow up of 30 months. At 4 years the disease free survival is 88% overall with 95% stage IA and IIA disease free, 92% stage IB, IIB, III and 53% stage IV disease. There was no difference between the two chemotherapy regimens. This study included a large number of patients and reported a high disease free survival, but all responding patients received radiotherapy. Only eight of 30 of our responding, previously untreated patients received radiotherapy.

A lower rate of response or relapse free survival than previously reported would be acceptable if most patients were spared radiotherapy and an alkylating agent and those who relapsed could be successfully salvaged. This strategy would be unacceptable if either there was a low second CR rate or if the late effects of second line therapy combining radiation and chemotherapy were major and involved a significant proportion of patients.

There are few reported series of the salvage rate with different regimens in Hodgkin's disease. Primarily resistant disease or patients who relapse within a year of primary therapy have a poor response to salvage therapy with a probability of less than 20% chance of remaining disease free 2 years from the salvage therapy (James *et al.*, 1990). In our series of 34 previously untreated patients there have been two deaths with a median follow up of 26.5 months (range 6–49 months). Of the seven patients who had primary resistant disease or initial Pr and then PD, there have been four who achieved Cr with ChlVPP (× 2), LOPP (× 1) and radiotherapy (× 1). The follow up from the time of diagnosis in these children are respectively 8, 8, 17 and 34 months. The remaining three patients did not respond to subsequent treatment and two have died, one is alive with disease.

Of the five children who relapsed, 2nd line ChlVPP has resulted in CR in all. It would therefore appear that most, if not all who relapse after an initial remission will achieve a second CR but only further follow up will show whether these children ultimately have as good an outcome as in historic series. As expected the efficacy of second line treatment is poorer in those who initially fail or progress on treatment. The high relapse rate in the children with CR(u) suggests that perhaps they should receive further treatment. Radford *et al.* described a group of 110 patients with mediastinal HD and showed that in those treated by chemotherapy alone, a residual abnormality was associated with a significantly higher relapse rate than those treated with a combined modality (Radford *et al.*, 1988). Two of the three patients treated with VEEP who achieved a CR(u) and subsequently relapsed did in fact receive mediastinal radiation and both relapsed in the abdomen. Our proposed strategy is that all patients with residual mediastinal disease should be biopsied and radiation withheld in the absence of histologically proven disease.

It is too early to draw firm conclusions from these results but further follow up will show whether or not salvageable relapse in a minority is an acceptable price to be paid for avoiding late sequelae in the majority.

### References

BARRETT, A., CIENNAN, E., BARNES, J., MARTIN, J. & RADFORD, M. (1990). Treatment of clinical stage I Hodgkin's disease by local radiation therapy alone. A UKCCSG study. *Cancer*, **66**, 670

BEHIENDT, H., VAN BUNNINGEN, B.N.F.M. & VAN LEEUWEN, E.F. (1987). Treatment of Hodgkin's disease in children with or without radiotherapy. *Cancer*, **59**, 1870.

CARBONE, P.P., KAPLAN, H.S., MUSSHOFF, K., SMITHERS, D.W. & TUBIANA, M. (1971). Report of the Committee on Hodgkin's disease staging. *Cancer Res.*, **31**, 1860.

CROWTHER, D. & LISTER, T.A. (1990). The Cotswolds report on the investigation and staging of Hodgkin's disease. *Br. J. Cancer*, **62**, 551.

DE LENA, M., MIELLO, E., BOZZI, D. & 5 others (1987). CHOP-B vs CEOP-B in poor prognosis non-Hodgkin's lymphoma: a randomised study. *Proceedings from Satelitte Symposium*, ECCO-4.

DONALDSON, S.S. & KAPLAN, H.S. (1982). Complications of treatment of Hodgkin's disease in children. *Cancer Treat. Rep.*, **66**, 977.

EKERT, H., WATERS, K.D., SMITH, P.J., TOOGOOD, I. & MAUGER, D. (1988). Treatment with MOPP or ChlVPP chemotherapy only for all stages of childhood Hodgkin's disease. *J. Clin. Oncol.*, **6**, 1845.

FRENCH COOPERATIVE (1988). High-dose *vs* low-dose radiation in childhood Hodgkin's disease: update of the French Cooperative Study. *Proceedings of SIOP*.

JAMES, N.D., KINGSTON, J.E., PLOWMAN, P.N. & 6 others (1990). Outcome of children with resistant and relapsed Hodgkin's disease. *Proceedings from British Oncological Association*, July 1991, p. 49.

JENKIN, D., CHAN, H., FREEDMAN, M. & 5 others (1982). Hodgkin's disease in children: treatment results with MOPP and low-dose, extended field irradiation. *Cancer Treat. Rep.*, **66**, 949.

NERI, B., CINI-NERI, G., BANDINELLI, M., PACINI, P., BARTALUCCI, S. & CIAPINI, A. (1989). Doxorubicin and Epirubicin cardiotoxicity: experimental and clinical aspects. *Int. J. Clin. Pharmacol. Ther. Toxicol.*, **27**, 217.

PEDERSEN-BJERGAARD, J., DAUGAARD, G., HANSEN, S.W., PHILIP, P., LARSEN, S.O. & RORTH, M. (1991). Increase risk of myelodysplasia and leukaemia after etoposide, cisplatin, and bleomycin for germ-cell tumors. *Lancet*, **338**, 359.

PERREN, T.J., SELBY, P.J., MILAN, S., MELDRUM, M. & MCELWAIN, T.J. (1990). Etoposide and adriamycin containing combination chemotherapy (HOPE-Bleo) for relapsed Hodgkin's disease. *Br. J. Cancer*, **61**, 919.

PUI, C.-H., BEHM, F.G., RAIMONDI, S.C. & ? others (1989). Secondary acute myeloid leukaemia in children treated for acute lymphoid leukemia. *N. Engl. J. Med.*, **321**, 136.

RADFORD, J.A., COWAN, R.A., FLANAGAN, M. & 4 others (1988). The significance of residual mediastinal abnormality on the chest radiograph following treatment for Hodgkin's disease. *J. Clin. Oncol.*, **6**, 940.

RADFORD, M., BARRETT, A., MARTIN, J. & ? others (1991). Treatment of Hodgkin's disease in children: study H.D.I. A report from the United Kingdom Children's Cancer Study Group. *Med. Ped. Oncol.*, **19**, 400.

ROBINSON, B., KINGSTON, J., NOGUEIRA COSTA, R., MALPAS, J.S., BARRETT, A. & MCELWAIN, T.J. (1984). Chemotherapy and irradiation in childhood Hodgkin's disease. *Arch. Dis. Child.*, **59**, 1162.

SANTORO, A., BONADONNA, G., VALAGUSSA, P. & 9 others (1987). Long-term results of combined chemotherapy – radiotherapy approach in Hodgkin's disease: superiority of ABVD plus radiotherapy versus MOPP plus radiotherapy. *J. Clin. Oncol.*, **5**, 27.

SELBY, P., PATEL, P., MILAN, S. & 9 others (1990). ChlVPP combination chemotherapy for Hodgkin's disease: long term results. *Br. J. Cancer*, **62**, 279.

SUTCLIFFE, S.B. (1987). Infertility and gonadal function in Hodgkin's disease. In *Hodgkin's Disease*, Selby, P. & McElwain, T.J. (eds), p. 339. Blackwell: Oxford.

TAYLOR, R.E. & HOMES, J. (1982). Etoposide as a single agent in advanced relapsed lymphomas. A phase II study. *Cancer Chemother. Pharmacol.*, **7**, 175.

WEINER, M.A., LEVENTHAL, B.G., MARCUS, R. & ? others (1991). Intensive chemotherapy and low-dose radiotherapy for the treatment of advanced-stage Hodgkin's disease in pediatric patients: a pediatric oncology group study. *J. Clin. Oncol.*, **9**, 1591.

WHITLOCK, J.A., GREER, J.P. & LUKENS, J.N. (1991). Epipodophyllotoxin-related leukemia. Identification of a new subset of secondary leukemia. *Cancer*, **68**, 600.

WORLD HEALTH ORGANIZATION (1979). *WHO Handbook for Reporting Results of Cancer Treatment*. WHO: Geneva.